# Inhibitory effects of cholinesterase inhibitor donepezil on the Kv1.5 potassium channel

**DOI:** 10.1038/srep41509

**Published:** 2017-02-13

**Authors:** Kai Li, Neng Cheng, Xian-Tao Li

**Affiliations:** 1 Department of Neurobiology, College of Life Science, South-Central University for Nationalities, Wuhan, 430074, China; 2 Graduate Institute of South-Central University for Nationalities, Wuhan, 430074, China

## Abstract

Kv1.5 channels carry ultra-rapid delayed rectifier K^+^ currents in excitable cells, including neurons and cardiac myocytes. In the current study, the effects of cholinesterase inhibitor donepezil on cloned Kv1.5 channels expressed in HEK29 cells were explored using whole-cell recording technique. Exposure to donepezil resulted in a rapid and reversible block of Kv1.5 currents, with an IC_50_ value of 72.5 μM. The mutant R476V significantly reduced the binding affinity of donepezil to Kv1.5 channels, showing the target site in the outer mouth region. Donepezil produced a significant delay in the duration of activation and deactivation, and mutant R476V potentiated these effects without altering activation curves. In response to slowed deactivation time course, a typical crossover of Kv1.5 tail currents was clearly evident after bath application of donepezil. In addition, both this chemical and mutant R476V accelerated current decay during channel inactivation in a voltage-dependent way, but barely changed the inactivation and recovery curves. The presence of donepezil exhibited the use-dependent block of Kv1.5 currents in response to a series of depolarizing pulses. Our data indicate that donepezil can directly block Kv1.5 channels in its open and closed states.

Alzheimer’s disease (AD) is an age-related neurodegenerative disease with pathological hallmarks, including extracellular amyloid plaques and intracellular neurofibrillary tangles^1^. Several cholinesterase inhibitors, such as tacrine, donepezil, rivastigmine and galantamine, are used as the clinical drugs to improve the cognitive impairment in early and mild stages of AD^2^. As one of the second generation of cholinesterase inhibitors, donepezil displays less adverse effects than earliest known cholinesterase inhibitors, such as physostigmine and tacrine^3^. Previous studies have indicated that donepezil generates beneficial effects in neuronal damage and cognitive deficits after ischemic insults^4^,^5^. Donepezil also displays anti-apoptotic effects against morphine-induced apoptosis in rat cerebral cortex and lumbar spinal cord^6^,^7^. Those data imply that donepezil can exert its action on multiple targets.

In excitable tissue, the functional expressions of various Kv channels are detected by many researchers^8^. Kv channels have a crucial role in the control of electrical signaling and sensitivity in neuronal and cardiac tissues^9^,^10^. Interestingly, published data show that cholinesterase inhibitors produce inhibitory effects on those channels in distinct preparations. For instance, rivastigmine inhibits the transient outward K^+^ current (IK(A)) and the delayed rectifier K^+^ current (IK(DR)) in acutely dissociated rat hippocampal pyramidal neurons^11^. Both bis(7)-tacrine and tacrine reduce the IK(A) in rat DRG neurons and currents through Kv4.2 channels expressed in *Xenopus oocytes*^12^. Similarly, studies indicate that donepezil blocks the IK (A) and IK (DR) in rat dissociated hippocampal neurons^13^,^14^. Moreover, donepezil-induced inhibition is detected in heterologously expressed hERG^15^ and Kv2.1^5^ channels in HEK293 cells. Noticeably, the prolonged QT interval and Torsade de Pointes arrhythmias are adverse side effects after treatment with donepezil^16^,^17^. The blocking effects of donepezil in human cardiac IK (DR) carried by hERG channels^18^ could contribute to side effects^15^. Rapid activating Kv1.5 channels are widely expressed in a variety of tissues and carry ultra-rapid delayed rectifier K^+^ current (IKur) which contributes to repolarization in human atrial cells^19^,^20^,^21^. In particular, it is demonstrated that Kv1.5 channels are responsible for one of the key components of IK (DR) in hippocampal neurons^22^,^23^. To date, however, there is little data regarding the possible action of donepezil on Kv1.5 channels. To address this issue, patch-clamp recording was conducted to explore the action of donepezil on heterologously expressed Kv1.5 channels in HEK293 cells.

## Materials and Methods

### Expression and mutation of Kv1.5 channels

All experiments were performed on Kv1.5 channels expressed in HEK293 cell lines. The methods used to culture cells have been described in our previous publication^24^. Briefly, the cells were grown in DMEM supplemented with 10% fetal calf serum, 100 U/ml penicillin and 100 ug/ml streptomycin.

Rat Kv1.5 in pEYFP-N1 vector (Clontech) was a gift provided by Dr. Len Kaczmarek (Yale University, the School of Medicine, New Haven, CT). Mutations to Kv1.5 channels were conducted with the QuikChange Site-directed Mutagenesis Kit (Stratagene, LaJolla, CA). Before electrophysiological study, transfection of Kv1.5 plasmids were carried out using Lipofectamine 2000 (Life Technologies, Bethesda, MD) according to the manufacturer’s protocol.

### Electrophysiology

The cells cultured on glass coverslips were removed from the incubator and mounted in a superfusion chamber. The patch-clamp experiment was undertaken using an Axopatch 200B amplifier and pClamp10 software (Molecular Devices, Sunnyvale, CA). Patch electrodes were fabricated from thin-walled borosilicate glass capillary by PC-10 puller (Narishige Instrument). The standard bath solution contained (in mM): 75 Na-gluconate, 70 NaCl, 5 KCl, 5 HEPES and 5 glucose, with pH adjusted to 7.4 using NaOH. Patch pipettes were backfilled with a high-K^+^ saline containing (in mM) 150 KCl, 5 HEPES, 5 EGTA, 5 Glucose, 5 Na_2_ATP. The pH of pipette solutions was adjusted to 7.3 with KOH. Data were filtered at 2 kHz (−3 dB) and sampled at 5 kHz for current recordings. Unless otherwise stated, a holding potential of −80 mV was used as the standard for all measurements. The compensations for membrane capacitances and 90% series resistances were routinely conducted during whole-cell recording. The stable Kv1.5 currents without run down after application of test pulses were selected for treatment with drug and further analysis. All measurements in this study were performed at room temperature (22 ± 1 °C).

### Analysis and statistics

Concentration-response curves of donepezil-induced inhibition of Kv1.5 channels were fitted by the Hill equation: *y = I*_min_ + (*I*_max_ − *I*_min_)/(1 + (IC_50_/*x*)^*h*^), where *I*_max_ and *I*_min_ are current with maximum and minimum, respectively, IC_50_ is the concentration for 50% block, and *h* is the Hill coefficient.

The single exponential *f* (*t*) = *A* exp (−*t* ⁄*τ*) + *A*_ss_, where *τ* is the time constant, *A* is the amplitude of the current component, and *A*_ss_ is the amplitude of the steady state current, was used to fit the obtained Kv1.5 current.

Data collected for building the activation and inactivation curve were fitted by a Boltzmann equation: *I/I*_max_ = 1/[1 + exp (*V*_1 ⁄ 2_ − *V *)/*k*], where *V*_1 ⁄ 2_ is the half-maximal activation potential for activation gate or the half-maximal inactivation potential for inactivation gate, *V* is the conditioning potential, and *k* describes the steepness of the curve.

All values are shown as mean ± S.E.M. Statistical significance of obtained results was evaluated using paired Student’s *t*-test or one-way analysis of variance (ANOVA). The statistical significance was set at *p* < 0.05.

## Results

### The blocking effect of donepezil on Kv1.5 currents

**Figure 1:**
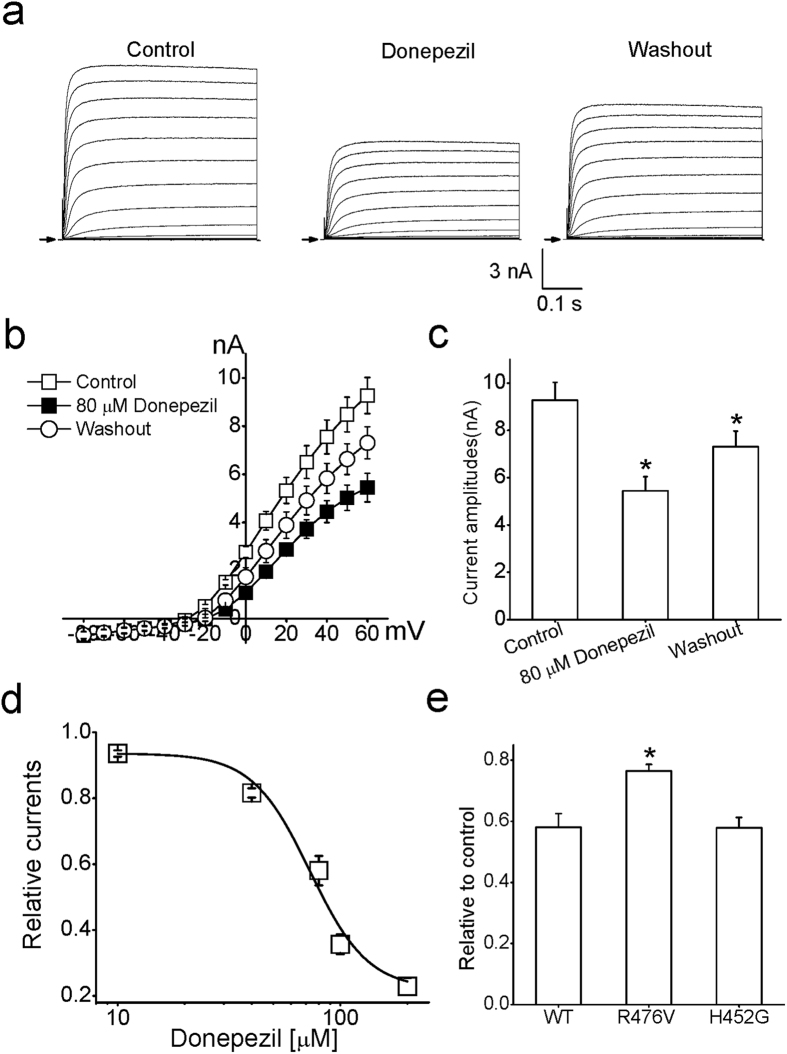
The blocking effect of donepezil on Kv1.5 currents. (**a**) Example traces of Kv1.5 currents were obtained in response to a 500 ms depolarizing pulse to potentials from −80 to +60 mV from a holding potential of −80 mV under control, 80 μM donepezil and washout conditions. Arrows denoted the zero-current level. (**b**) Current-voltage (*I*–*V*) relationships of Kv1.5 currents with and without donepezil (*n* = 11). (**c**) Bar graph of statistical analyses of block by donepezil in Kv 1.5 channels at +60 mV. *p < 0.05 compared with the control group. (**d**) The concentration-response relations of donepezil action on Kv1.5 currents. Data points with various concentration donepezil were fitted by a Hill equation (*n* = 6). (**e**) Mutant R476V but not H452G reduced the affinity of donepezil to Kv1.5 channels. Collected raw data were normalized to the control, and then were plotted with respect 80 μM donepezil (*n* = 5). Unless otherwise noted, WT indicates wild type in all figures. *p < 0.05 compared with the wild type group.

After transfection of Kv1.5 channel plasmids in HEK293 cells, whole-cell recording was carried out to assess the effect of donepezil on corresponding currents. In the presence of 80 μM donepezil, a significant decrease was detected in Kv1.5 currents, which were elicited by depolarizing the cell from a holding potential of −80 mV to a test potentials ranging from −80 to +60 mV. This action was partially reversed following washout of the extracellular donepezil (Fig. 1a). The current-voltage (I–V) relationships of Kv1.5 currents in control, donepezil and washout are shown in Fig. 1b. The significant alterations of current amplitudes among the control, donepezil and washout were detected at different voltages between 0 mV to +60 mV (*n* = 11, ANOVA, *p* < 0.05). In Fig. 1c, the statistical analyses of block by donepezil at +60 mV are shown as the bar graph. Another cholinesterase inhibitor tacrine, however, barely affected the Kv1.5 currents, even in the higher concentration (200 μM, data not shown).

The concentration-dependent action of donepezil on these currents was explored, and the concentration-response relationships were constructed in Fig. 1d. The normalized data were well fitted by Hill equation with the IC_50_ value of 72.5 ± 0.5 μM and Hill coefficient of 3.4 ± 0.8 (*n* = 6). Previous reports have revealed that the residue 476 is crucial for the action of nifedipine^25^ and external pH on Kv1.5 channels^26^. The arginine at the position 476 equivalent to the presumed TEA binding site in *Shaker* (T449) and Kv2.1 (Y380) is located within the conduction pathway of Kv1.5^25^,^27^. Thus, we measured the effect of donepezil on mutant R476V of this channel in which arginine at position 476 was replaced with valine. As shown in Fig. 1e, the effect of 80 μM donepezil on R476V variant currents was significantly weaker than that on wild type currents (*n* = 5, Student’s *t*-test, *p* < 0.05), suggesting that the outer mouth of Kv1.5 channels is the binding site of this chemical. Nevertheless, substitution of the histidine with a glycine at position 452, another TEA binding site^26^,^28^, produced a negligible effect after perfusion of 80 μM donepezil (*n = *5, Student’s *t*-test, *p* > 0.05).

### The action of donepezil on the activation of Kv1.5 channels

**Figure 2:**
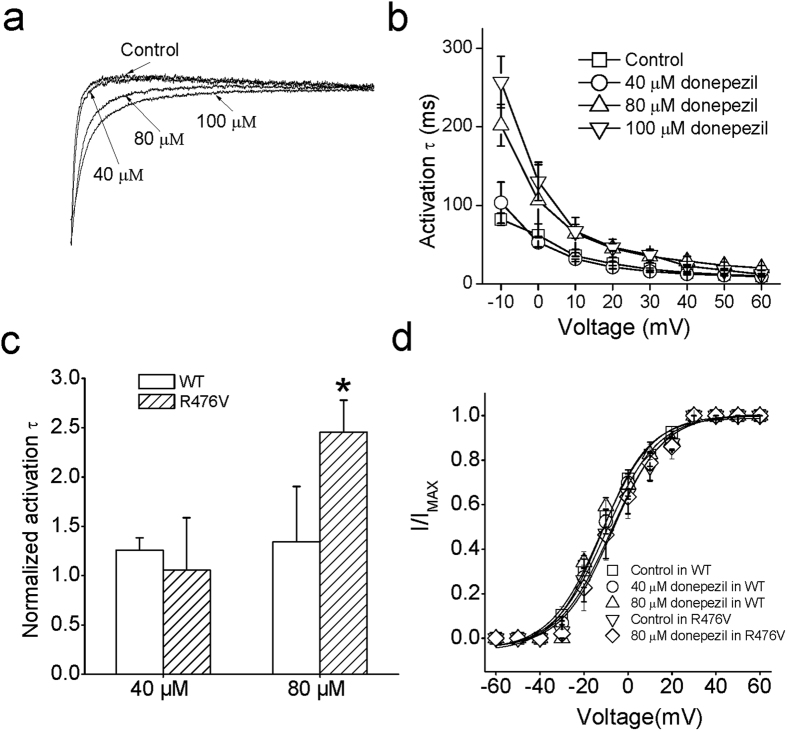
Alteration of Kv1.5 activation by donepezil. (**a**) A plots of the raising phases of currents traces in control, 40, 80 and 100 μM donepezil at +10 mV. The current amplitude was normalized to that of the steady-state current. (**b**) The activation traces were well fitted by a single exponential function and activation time constant (τ_act_) was plotted against the test voltages. Exposure to 80 and 100 μM donepezil lead to an enhancement in τ_act_ at potential more negative than 20 mV. (*n* = 5). (**c**) Bar graph showed that mutant R476V resulted in a delay in activation time course of treatment with 80 μM rather than 40 μM donepezil. All data points were normalized to those in respective control (*n* = 5). (**d**) Normalized data (*I/I*_max_) were plotted against conditioning prepulse potentials and fitted with a Boltzmann equation (*n* = 6). Activation curves were plotted with donepezil in wild type and R476V channels. No significant difference between wild type and mutant R476V was detected, regardless of the addition of donepezil.

Representative current traces after treatment with varying concentration donepezil are shown in Fig. 2a. As shown in Fig. 2b, it was clearly implied that external application of 80 μM donepezil led to a significant delay in activation of Kv1.5 currents compared with the control. For example, the activation time constant (τ_act_) at −10 mV was 80.3 ± 7.8 ms in control and became 202 ± 26.6 ms in 80 μM donepezil (*n* = 5, Student’s *t*-test, *p* < 0.05). But no significant effect was observed after treatment with 40 μM donepezil. The value of τ_act_ with 40 μM donepezil at −10 mV was 103.5 ± 26.2 ms, which was little different from that value in the control (*n* = 5, Student’s *t*-test, *p* > 0.05). Interestingly, the outer pore of mutant R476V also enhanced the efficacy of donepezil-decreased τ_act_ at a concentration of 80 μM rather than 40 μM (Fig. 2c; *n* = 5, Student’s *t*-test, *p* < 0.05). As seen in Fig. 2b, the values of τ_act_ decreased with membrane depolarization and this trend was unaffected during introduction of donepezil.

To examine whether donepezil affects the activation curves, the currents were recorded at the test potential of −40 mV following a conditioning prepulse of various potentials from a holding potential of −80 mV. The peak values of tail current were normalized by comparing to its maximal value and then fitted by a single Boltzmann equation for building activation curves. the value of *V*_1/2_ was -12.1 ± 1.2 mV in control, -10.1 ± 1.4 mV in 40 μM and -12.3 ± 1.7 mV in 80 μM donepezil, indicating that this molecule did not change the activation gating of Kv1.5 channels (Fig. 2d; *n* = 6, ANOVA, *p* > 0.05). In addition, mutation of R476V produced a negligible action on the activation curves after perfusion of 80 μM donepezil (*n* = 5, ANOVA, *p* > 0.05).

### The effect of donepezil on the deactivation kinetics of Kv1.5

**Figure 3:**
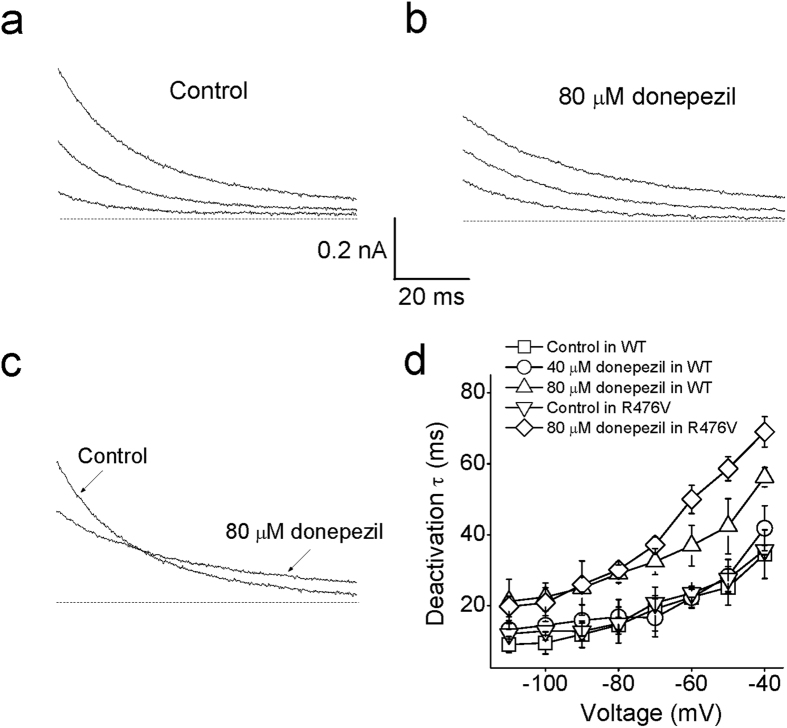
The deceleration effect of donepezil on deactivation time course of Kv1.5 current. (**a**) The representative traces of Kv1.5 deactivation tail currents in control were exhibited. The tail currents were in response to a long 500 ms voltage steps to potentials from −100 mV to −40 mV after a conditioning prepulse to +40 mV. Dashed lines donated the zero current level. (**b**) The typical traces of Kv1.5 currents after introduction of donepezil. (**c**) The crossover phenomena were observed after superimposing the control trace to the trace with donepezil. (**d**) Statistical analysis of donepezil’s effect on deactivation time constant at +40 mV (*n* = 5). *p < 0.05 compared with the control group.

At a holding potential of −80 mV, the deactivation tail currents were elicited by a series of 500 ms test pulses from −110 mV to −40 mV after a conditioning prepulse of +40 mV. The representative superimposed tail currents before and after application of 80 μM donepezil are shown in Fig. 3. These currents were well fitted by a single exponential function to obtain the deactivation time constants (τ_deact_). At −40 mV, control tail currents decreased with a time constant of 34.5 ± 6.8 ms and reached the steady state at the end of test potential. Perfusion of 80 μM donepezil slowed the tail current deactivation with a 56.2 ± 2.7 ms of τ_deact_ (*n* = 6, Student’s *t*-test, *p* < 0.05), and no significant difference was measured in the presence of 40 μM donepezil compared to untreated controls at −40 mV (*n* = 6, Student’s *t*-test, *p* > 0.05). In the presence of 80 μM donepezil, interestingly, the R476V variant significantly prolonged τ_deact_ to 69.1 ± 2.7 ms, which was larger than that in wild type channels (*n* = 5, Student’s *t*-test, *p* < 0.05). The statistical analyses of τ_deact_ against test potentials were present in Fig. 3d. Apparently, the value of τ_deact_ under control conditions increased at the potentials between −100 mV to −40 mV (ANOVA, *p* < 0.05). In addition, superfusion of 80 μM but not 40 μM donepezil enhanced the τ_deact_ compared with the control (*n* = 6, Student’s *t*-test, *p* < 0.05), suggesting that this chemical slowed the decay of deactivation currents. Therefore, it is reasonably expected to observe the “crossover” of current traces during measurement. A typical example of the current crossover before and after application of 80 μM donepezil is illustrated in Fig. 3c. It implies that donepezil must dissociate from its binding site before Kv1.5 channels can close. Thus, donepezil may work as an open channel blocker in Kv1.5 channels.

### The action of donepezil on inactivation

**Figure 4:**
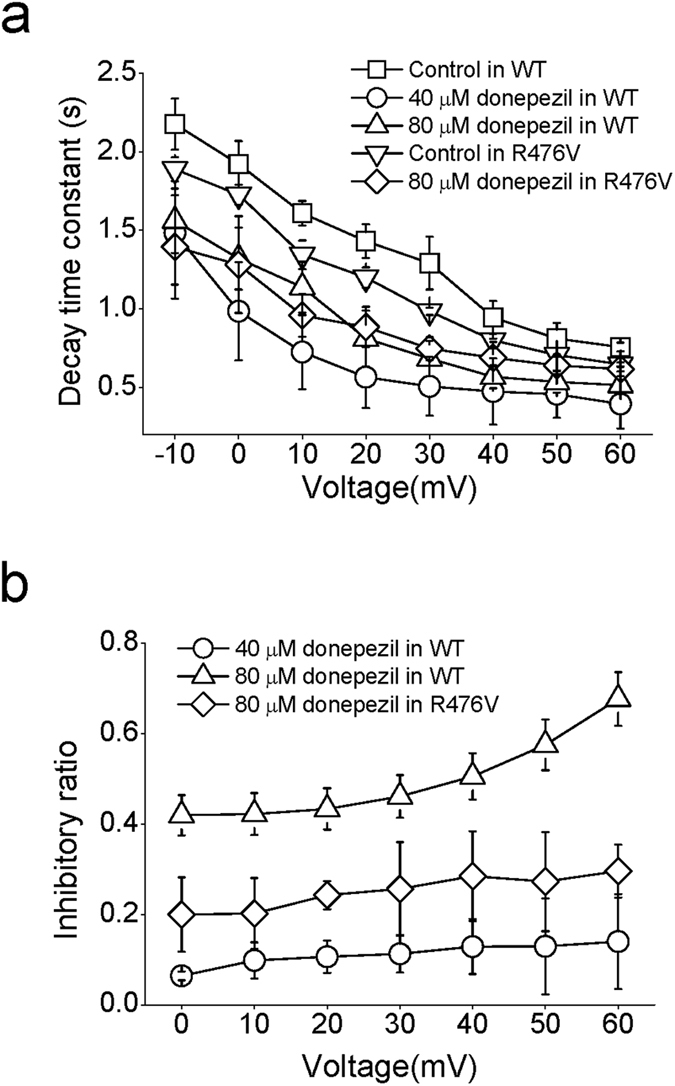
The effect of donepezil on the inactivation time constants of Kv1.5 currents. (**a**) A long 15 s voltage pulse to +60 mV from the holding potential of −80 mV were undertaken to assess the Kv1.5 inactivation. The inactivation time constants (τ_inact_), which were calculated by fitting decay currents using a exponential equation, were plotted against the test potential. Introduction of donepezil resulted in the accelerations of τ_inact_ in a voltage-dependent way (*n* = 5). (**b**) The graph showed that the donepezil-mediated block of Kv1.5 channels was voltage dependent (*n* = 6).

To detect the action of donepezil on Kv1.5 inactivation, a series of 15 s test pulses ranging from −10 mV to +60 mV from a holding potential of −80 mV were carried out. The elicited currents were well fitted using a exponential function to obtain the inactivation time constant (τ_inact_), and then plot of relationship between τ_inact_ and membrane voltage was constructed in Fig. 4a. At -10 mV, the value of τ_inact_ was 2.2 ± 0.2 s in control and became 1.5 ± 0.2 s in 80 μM donepezil, suggesting a 31.8% of acceleration in current decay compared with the control (Fig. 4a; *n* = 5, Student’s *t*-test, *p* < 0.05). Clearly, exposure to donepezil substantially accelerated the Kv1.5 current inactivation. In addition, mutant R476V produced a reduction in τ_inact_ compared to wild type control as well (*n* = 5, Student’s *t*-test, *p* < 0.05).

The voltage-dependent blocking effects of donepezil were evaluated using data obtained from above long pulses. As seen in the plot of the inhibitory ratio against test potentials (Fig. 4b), the blockage of donepezil on Kv1.5 currents increased with depolarization. At +50 mV, 80 μM of donepezil inhibited Kv1.5 currents by 56.5% compared with the control (*n* = 6, Student’s *t*-test, *p* < 0.05). At +10 mV, a decrease of 42.2% in these currents, however, was produced by identical concentration of donepezil, implying a voltage-dependent blockage (*n* = 6, Student’s *t*-test, *p* < 0.05). However, both mutant R476V (*n* = 6) and 40 μM donepezil (*n* = 6) failed to generated the significant alteration in inhibitory ration over the voltage range tested (ANOVA, *p* > 0.05).

**Figure 5:**
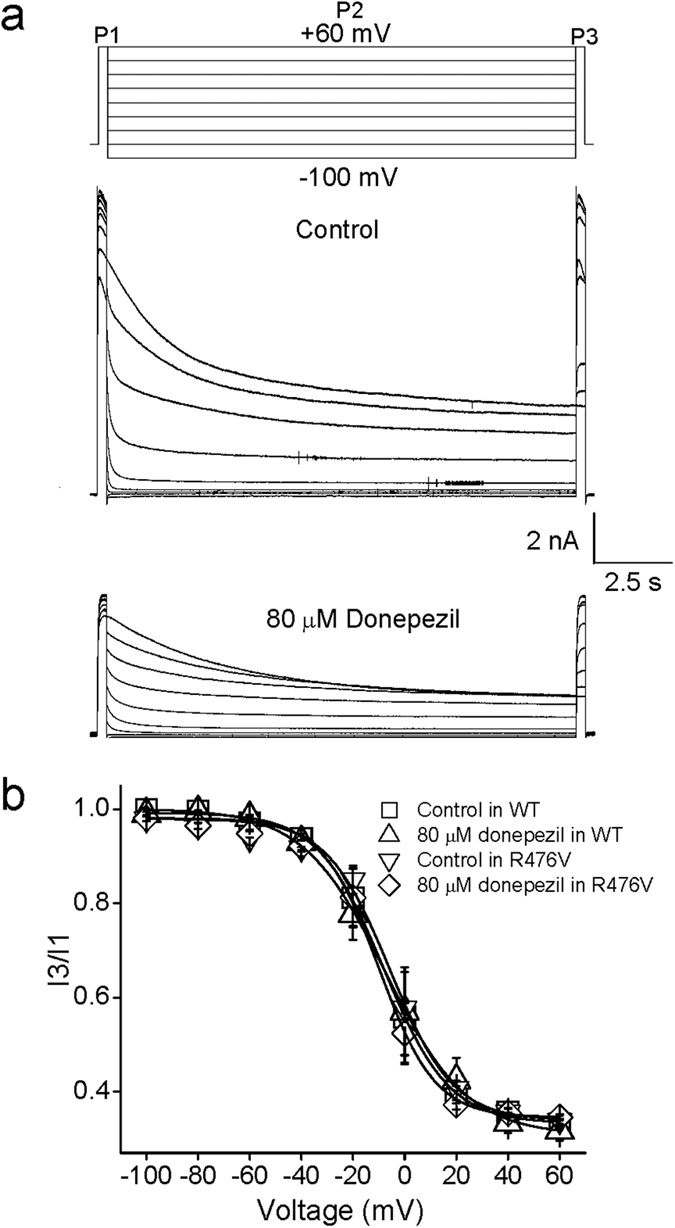
Donepezil failed to change the inactivation curve. (**a**) The schematic illustrated the three-pulse protocol which was taken to generate the currents for constructing the inactivation curve (top panel). Superimposed current records were present for control (middle panel) and application of donepezil (bottom panel). (**b**) The currents (I3) in response to the test pulse (P3) were normalized to currents (I1) elicited by conditioning prepulse (P1), and then data points (I3/I1) were well fitted using a Boltzmann equation to obtain inactivation curves. Both mutant R476V and donepezil unaltered these curves (*n* = 6).

A three-pulse (3P) protocol described in previous publication, which could avoid cumulative inactivation^29^, was employed for examining inactivation kinetics of Kv1.5 currents. As shown in top panel of Fig. 5a, a voltage step to potential of +60 mV was the first pulse (P1), which was regarded as a control for accumulation of inactivation. The inactivation currents were evoked by second voltage pulses (P2) with long duration (15 s) to potentials ranging from −100 and +60 mV. Currents taken from the final pulse (P3) to test potential of +60 mV were normalized to those from P1, and then normalized data were plotted against P2 potentials and fitted with a Boltzmann equation. Example current traces recorded using above protocol are present in Fig. 5a. As shown in Fig. 5b, inactivation of Kv1.5 currents actually occurred at potentials ranging from −60 mV to +60 mV. In accordance with previous report^30^, the inactivation of Kv1.5 currents appeared to be incomplete even after a 15 s inactivation duration. Data points in control were well fitted with a single Boltzmann with *V*_1/2_ value of −8.1 ± 0.7 mV and *k* value of 12.6 ± 0.6 mV. After bath application of 80 μM donepezil, the *V*_1/2_ and *k* showed no change, measuring −7.5 ± 0.8 mV and 13.8 ± 0.8 mV, respectively (*n* = 6, Student’s *t*-test, *p* > 0.05). Mutant R476V barely affected the inactivation curves of Kv1.5 channels with and without 80 μM donepezil.

### Use-dependent block of Kv1.5 by donepezil

**Figure 6:**
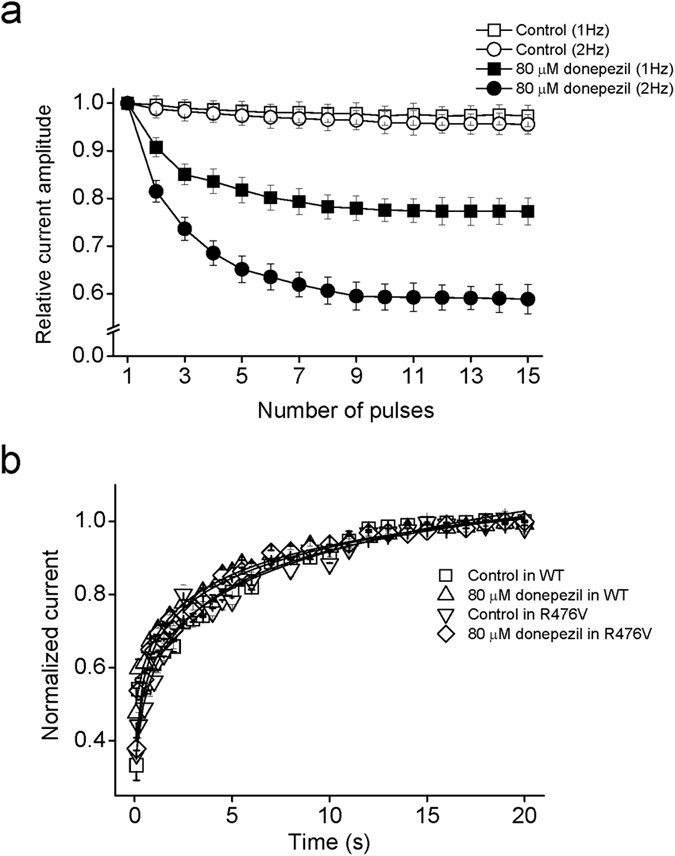
The effects of donepezil on the use-dependent block and recovery of Kv1.5 currents. (**a**) Plot of use-dependent changes in relative peak Kv1.5 currents with and without 80 μM donepezil (*n* = 5). At a holding potential of −80 mV, Kv1.5 currents were evoked in response to 250 ms voltage pulses applied at the frequency of 1 and 2 Hz to a potential of +30 mV. (**b**) To evaluate the action of donepezil on recovery of Kv1.5 channels, currents were evoked by a 100 ms test pulse to +40 mV after a 3 s conditioning prepulse to +40 mV with various intervals from 100 ms to 20 s. Normalized recovery currents were plotted against time intervals for wild type and R476V variant before and after application of 80 μM donepezil. Data points were well fitted using a mono-exponential function to obtain the smooth curves (*n* = 6).

Previous studies have shown that some drugs block Kv channels in a use-dependent manner, in which the rate of stimulation affects the degree of drug block^31^. In order to explore use-dependent block of Kv1.5 by donepezil, we recorded macroscopic currents which were elicited using 15 repetitive test pulses to +30 mV at two different frequencies, 1 and 2 Hz. Normalized peak current amplitudes at different frequencies were plotted as a function of the pulse number (Fig. 6a). In the absence of donepezil, the peak amplitude of Kv1.5 currents decreased by 2.7 ± 0.2% at 1 Hz and 4.4 ± 0.2% at 2 Hz (*n* = 5). In the presence of 80 μM of donepezil, the peak amplitude of Kv1.5 currents was suppressed by 22.7 ± 2.8% and 41.1 ± 3.1% at 1 Hz and 2 Hz, respectively (*n* = 5). Apparently, Kv1.5 channels are substantially inhibited by donepezil when channels are repeatedly shuttled between positive and negative potentials, suggesting a use-dependent block.

### The effect of donepezil on recovery of Kv1.5 from inactivation

We finally examined the actions of donepezil on the recovery rate of Kv1.5 channels using a dual pulse protocol. Briefly, the 3 s prepulse to potential of +40 mV initiated inactivation of Kv1.5 channel, and then voltage step to potential of −80 mV with a variable time interval ranging from 50 ms to 20 s induced different potency of recovery. At last, the peak currents during each test pulse to +40 mV were normalized with respect to maximal currents (*I/I*_max_) for assessing the extent of recovery. Normalized data were plotted against time interval and fitted with a single exponential equation. The recovery time constant (τ_recor_) acquired after fitting is 3.9 ± 0.3 s in control and 4.7 ± 0.6 s in 80 μM donepezil, suggesting that donepezil did not change the recovery of Kv1.5 currents (Fig. 6b; Student’s *t*-test*, n = *6, *p* > 0.05). Mutant R476V unaffected the recovery curves as well.

## Discussion

In the present study, we showed a result that donepezil, a potent inhibitor of acetylcholinesterase, blocked Kv1.5 channels expressed in HEK293 cells in voltage- and concentration-dependent ways. Earlier publications unfold that chemicals, such as nifedipine^24^ and celecoxib^32^, have an inhibitory effect on both closed and open states of Kv2.1 channels. Similarly, our data revealed that donepizil also worked as a blocker of Kv1.5 channels through above two manners. A deceleration of activation time course by external application of donepezil was consistent with the behavior of the closed-channel blocker, which was noted in previous data^33^. Nevertheless, there is more evidence in this study that supports the idea of open channel binding of donepezil to Kv1.5 channels. For instance, the donepezil-mediated acceleration in decay rate of Kv1.5 current inactivation is in line with expedited inactivation by open-channel blocker^34^,^35^. Moreover, the phenomenon of crossover due to the deactivaton delay induced by open channel blocker^36^ was apparently evident in our data.

Our data reveal that the inhibition of Kv1.5 channels by donepezil are use-dependent, which reflects the dynamics of the on and off rates for drug binding. Interestingly, literature data also show that Kv1.5 channels are use-dependent block by several other drugs. For instance, this channel can be blocked by AVE0118^37^, sertraline^38^ and natural flavone acacetin^39^ in a use-dependent way. As a result, AVE0118, a novel antiarrhythmic drug, may exert more action during the tachycardia than normal heart rates^37^. Thus, it suggests that state-dependent affinities are essentially involved in the block-associated proarrhythmia^40^.

The potency of donepezil block was substantially reduced in mutant R476V, showing that its binding site located in outer pore of Kv1.5 channels. This molecule may be protonated at physiological pH^14^ and the removal of arginine, a residue with positive charge, should lead to an enhancement of donepezil’s effect due to the alteration of electrostatic interaction. The opposite observation, however, suggests that donepezil could act on R476V variant in its uncharged form. As seen in Fig. 4, mutation in the conduction pathway of Kv1.5 can also ameliorate voltage-dependent block of this chemical.

Interestingly, the mutation at position 476 equivalent to the TEA binding site resulted in an enhancement rather than attenuation in donepezil-induced delay of Kv1.5 channel activation. This observation supports the concept that R476 is not a site for directly regulating the activation time course. Similarly, Outer pore mutations of R476V also potentiated the deceleration effect of donepezil in the deactivation time course. Those data suggest that there exist multiple binding sites of donepezil in Kv1.5 channels, coinciding with the Hill coefficient of 3.4 calculated from the concentration-response curve. Apparently, it also indicates that an allosteric mechanism is involved in the regulation of Kv1.5 channels by donepezil.

Our data revealed that another cholinesterase inhibitor tacrine did not exert any action on macroscopic Kv1.5 currents (data not shown). The exact causes for the different effect between tacrine and donepezil on Kv1.5 currents remain unknown for us. Interestingly, bis(7)-tacrine, a dimeric acetylcholinesterase inhibitor with two tacrine molecules linked by a heptylene chain^41^, has much higher affinity in blocking Kv4.2^12^ and Kv1.2^42^ channels compared with tacrine. The distinct structure in those chemicals could result in varying sensitivity to Kv channels.

Yu *et al*. reported that donepezil suppressed the delayed rectifier K^+^ current (IK(DR)) in rat dissociated hippocampal neurons with higher IC_50_ values of 78 μM^13^. However, Solntseva *et al*. revealed that the IC_50_ values of donepezil-mediated block in above currents were 8.9 μM^14^. The exact causes for the differences in those reports are unknown and could be attributed to the distinct methods in stimulation protocol and pipette solution^14^. Our data revealed that the IC_50_ value of 72.5 μM for donepezil block of Kv1.5 channels is close to that value for IK(DR) reported by Yu *et al*. Interestingly, heteromeric channels assembled by Kv channel subunits are detected in the central nervous system and are responsible for diversity of function and sensitivity to various signal^43^. Kv1.5 has been shown to form heteromultimeric complexes with Kv1.2^44^ or Kv1.3^45^ in neural tissues. Therefore, it is possible that the assembly of Kv1.5 with other Kv channel subunits could result in the varying sensitivity to donepezil in neural tissues, including hippocampal neurons expressing Kv1.2^46^ and Kv1.3^22^.

Usually, the acidification may occur in some pathological conditions such as ischemia and hypoxia in cardiac^47^ and neural tissues^48^. As mentioned above, there exist both charged and uncharged forms of donepezil^14^, and the latter can cross the lipid membrane and reach the internal mouth of Kv1.5 channels. Under acidification conditions, donepezil may be potentially protonated and thus positively charged drug may enter deeply into the channel pore in a voltage-dependent way. Therefore, it is possible that the acidification induced by pathological conditions potentiates the effect of donepezil on Kv1.5 channels. Published data provide related evidence for this notion. For example, Wang *et al*. reported that low pH increased the extent and speed of dofetilide-induced block in hERG currents^49^.

Taken together, our data disclosed that donepezil inhibited the delayed rectifier Kv1.5 channels in both voltage-dependent and concentration-dependent ways. In addition, this molecule exerted an action on channel kinetics, such as activation and deactivation. The use-dependent block mediated by donepezil could exert a strong inhibition in Kv1.5 channels under appropriate conditions. The presence of donepezil exhibits the blocking effects on Kv1.5 channels by binding to both its open and closed states.

## Additional Information

**How to cite this article:** Li, K. *et al*. Inhibitory effects of cholinesterase inhibitor donepezil on the Kv1.5 potassium channel. *Sci. Rep.* **7**, 41509; doi: 10.1038/srep41509 (2017).

**Publisher's note:** Springer Nature remains neutral with regard to jurisdictional claims in published maps and institutional affiliations.
